# An innovative technique and laboratory protocol for CO_2_ storage in water disposal wells and monitoring asphaltene deposition

**DOI:** 10.1038/s41598-025-32916-9

**Published:** 2025-12-25

**Authors:** Ehsan Jafarbeigi, Shahab Ayatollahi

**Affiliations:** 1 Department of Chemical and Petroleum Engineering, Sharif University of Technology, P.O. Box 11155 − 9465, Tehran, Iran; 2 Department of Chemical and Petroleum Engineering, Ilam University, Ilam, Iran

**Keywords:** CO_2_ storage, Oil/Water emulsions, Asphaltene precipitation, CO_2_ injection, Water disposal zone, Chemistry, Energy science and technology, Engineering, Environmental sciences

## Abstract

Injecting CO_2_ into water-disposal wells is a promising strategy for geological carbon storage. However, this process can destabilize asphaltenes in residual oil blobs—primarily of the emulsified type – trapped within trapped in the porous rock, leading to precipitation that threatens storage integrity and operational safety. This study introduces a novel high-pressure laboratory apparatus and protocol designed to directly quantify asphaltene precipitation during CO_2_ injection into oil-in-water emulsions, which represent water-flooded formations. The system operates at reservoir-relevant conditions (up to 11,000 psi and 210 °C) and utilizes in situ near-infrared (NIR) light transmission to monitor asphaltene precipitation in real-time. Additionally, this research investigates the behavior of the oil-in-water emulsion (EM) phase as the medium hosting CO_2_ gas under different conditions. Quantitative results, expressed as the percentage reduction in NIR transmission, showed that asphaltene precipitation was minimized to 0.8% under optimal conditions (2DSW, 120 °C, 50 mol% CO_2_), compared to a peak of 25.1% in the worst-case scenario (FW, 30 °C, 35 mol% CO_2_). Regarding the CO_2_ injection rate, less asphaltene precipitation occurred at higher injection rates. In this case, crude oil vaporized in the EM phase at high CO_2_ injection rates (above 35 mol%), resulting in fewer crude oil droplets available to interact with CO_2_. Notably, EMs prepared with twice-diluted seawater (2DSW) exhibited the least asphaltene precipitation, a finding strongly correlated with lower oil/water interfacial tension. Overall, the developed protocol provides a critical tool for screening and de-risking CO_2_ storage sites in water-disposal zones by enabling accurate prediction of asphaltene-related damage.

## Introduction

To promote a more environmentally sustainable approach to reservoir development and carbon dioxide storage, it is essential to adopt innovative methods. Currently, significant challenges related to environmental degradation and the depletion of finite energy resources, particularly fossil fuels, have emerged^1,2^. In response, the European Union has prioritized carbon capture and sequestration and carbon capture and utilization in its Strategic Energy Technologies plan, as outlined in various research and innovation roadmaps^3,4^. Properly managing the storage of carbon dioxide in deep geological formations can significantly mitigate the risk of environmental contamination^5,6^. Under certain conditions, carbon dioxide can extract lighter components from oil, altering its properties. This extraction leads to a decrease in the solubility of heavier components, such as asphaltenes, which can subsequently precipitate out of the oil^7–9^. In this regard, asphaltene deposition is a severe flow assurance issue to oil and gas production that costs the industry a remarkable loss. Generally, asphaltene is not soluble in paraffinic solvents with low molecular weight, including n-heptane and n-pentane^10–15^. At first, asphaltene is stable in the crude oil and if any disturbance to this stability occurs, such as pressure depletion because of production drawdown, asphaltene begins to precipitate^16–23^. According to the classical colloidal model^24^, the asphaltene is in the micelle core surrounded by resin molecules^25^. The colloidal stability of crude oil during the oil production process is fragile and easily impacted by solution gas release and changes in pressure and temperature^26–30^. Generally, the stability of the crude oils colloidal system is highly sensitive to these factors, which can trigger a sequence of asphaltene aggregation, beginning with accumulation, progressing to flocculation, and culminating in precipitation^31^. These deposition phenomena pose significant flow assurance challenges, ultimately reducing injectivity and elevating operational costs^32–35^. It is worth noting, chemical inhibition is a primary strategy to manage asphaltene precipitation in production systems. Common classes of inhibitors act as dispersants by adsorbing onto asphaltene nanoaggregates and preventing their flocculation through steric repulsion^36–39^. Generally, water injection is a widely adopted oil recovery method, with low-salinity water flooding emerging as an improved technique for enhancing recovery efficiency^40–48^. A common consequence of this practice, however, is high water production, which presents a major operational challenge. Interestingly, the resulting produced water offers a potential opportunity; the injection wells and disposal aquifers used for this water can be repurposed for carbon sequestration near oil fields^49–51^. In this regard, a critical complication arises because this disposed water often contains a microemulsion of residual oil droplets. The introduction of CO_2_ into this environment can destabilize these droplets, creating a significant risk for asphaltene precipitation and jeopardizing the sequestration process^52–54^. Mokhtari et al. (2019) examined the effect of injected brine salinity on asphaltene instability. They indicated that in low salinity, the existence of the polarity of the oil-brine interface is effective in the adsorption of asphaltenes^55^. Shahsavani et al. (2021) investigated how an emulsified aqueous phase in synthetic oils influences asphaltene precipitation. They analyzed the impact of ions on the interaction between asphaltenes and the oil/water interface through interfacial rheology and droplet size measurements. Their findings revealed that higher valence ions, such as Fe^3+^, significantly reduced the size of water droplets in the emulsion and led to an increased retention of asphaltenes^56^. Tajikmansori et al. (2023)^57^ examined how the physicochemical characteristics of asphaltenes and resins, along with the concentration and type of dissolved ions (such as MgCl_2_, CaCl_2_, and Na_2_SO_4_) in the aqueous phase, influence interfacial interactions. In this regard, they evaluated the properties of asphaltene molecules using zeta potential measurements. Their findings indicated that resin molecules quickly occupy the interface due to their smaller relative molecular mass and size. Additionally, they found that the negative polar sites on the structures of asphaltenes and resins exhibited greater activity compared to the positive sites. In the past, the EOR performed by immiscible CO_2_ injections is especially proposed for its effective sweep efficiency, oil-viscosity reduction, oil-swelling effect, low-pressure requirements, and significant reduction of greenhouse gas emissions^58,59^. Peysson et al. (2014)^60^ investigated gas injection into wells during carbon dioxide storage operations in saline aquifers. They stated that the injection of gas in the saline aquifers may lead to salt precipitation and drying near the wellbore. Wang et al. (2018)^61^ investigated the likelihood of asphaltene depositions under carbon dioxide injections at different reservoir conditions. Their observations indicated that carbon dioxide concentration is the main factor to consider in asphaltenes deposition. Indeed, the thermodynamic conditions and gas injection fraction at which the carbon dioxide flooding is performed which is very important in the investigation of asphaltene deposition. Cho et al. (2019)^62^ investigated the impacts of formation damage caused by asphaltene deposition on three-phase hysteretic models for the prediction of coupled carbon dioxide EOR and storage performance. The results showed that formation damage by asphaltene deposition caused a 9% decrease in oil recovery and a 14% increase in water production compared to the model without asphaltene deposition. Hajiabadi et al. (2021)^63^ investigated the main factors affecting carbon dioxide injection in deep saline aquifers to identify gaps. They also examined the available analytical and numerical mathematical models to estimate the maximum stable injection pressure and pressure build-up. On the other hand, they noted that the primary debates concerning analytical models revolve around the overall configuration of the CO_2_–brine interface, which arises from either overlooking or making assumptions about CO_2_ compressibility, the mutual solubility of CO_2_ and water, and drying effects. Conversely, their study’s findings indicated that models designed to forecast waterflooding processes are unable to accurately assess CO_2_ injectivity because of the distinct characteristics of CO_2_. In another study, Yusof et al. (2022)^64^ investigated the quantification of the change in carbon dioxide injection caused by the solubility between the formation water and carbon dioxide on a laboratory scale through the coreflooding test. The salinity of injected brine was in the range (6000 ppm-100000 ppm). The results showed that there is a direct correlation between the intensity of injection changes caused by salt precipitation and salinity. They showed that as the salinity of the brine increases, the interference with carbon dioxide injection increases (from 6% to 26.7%). As delineated in Table 1, conventional technologies for determining asphaltene deposition are predominantly tailored for the analysis of dead oils with solvents or stable crude oil systems. A significant and persistent gap in the literature is the lack of a methodology for the direct and quantitative measurement of asphaltene precipitation within dynamic, high-turbidity oil-in-water emulsions under reservoir-relevant conditions. This specific scenario is critically important for accurately assessing flow assurance risks in water-disposal zones and water-flooded reservoirs, where residual oil exists as dispersed droplets. The present study directly addresses this technological gap. The innovation of our apparatus is not a mere incremental improvement but a targeted solution engineered for a previously unquantifiable problem. In this regard, while established tools such as Visual Cells and Quartz Crystal Microbalances (QCM)^65^ are designed for bulk crude oil or surface deposition studies, our apparatus is specifically engineered to investigate oil-in-water emulsions. This is crucial for simulating the environment of water-flooded reservoirs, where the interaction between injected fluids (e.g., CO_2_) and the residual oil occurs at the droplet interface—a complex scenario where traditional methods are inadequate. Also, the LUMiSizer^66^ is indeed a tool for analyzing emulsion stability, typically by measuring phase separation kinetics under centrifugal force. However, its standard operational range is often limited to near-ambient pressures and does not typically accommodate the simultaneous injection of high-pressure gases like CO_2_, which is the core of our investigated process.

**Table 1:** Comparison of asphaltene deposition measurement methods, perspectives, and limitations.

Method	Principle of Measurement	Key limitations and benefits	References
This study	Measures decrease in light transmission/scattering due to asphaltene precipitation.	Uniquely enables quantitative analysis in highly turbid, dark crude oils. Key advantages include a multi-purpose cell, a powerful halogen light source enabling transmission through > 3 mm of oil.	This study
NIR Spectroscopy	Measures decrease in light transmission/scattering due to asphaltene precipitation.	Struggles with highly turbid or dark crude oil samples, limiting quantitative analysis.	^67^
Visual/Organic Deposition Cell	Direct visual observation of precipitation in a PVT cell.	Cannot provide quantitative data; ineffective for high-turbidity emulsions and dark oils.	^68^
Pendant Drop	Detects a sudden change in interfacial tension (IFT) at the onset point.	An indirect method; requires interpretation of IFT curves and may not detect early precipitation.	^69^
Electrical Conductivity	Monitors increase in conductivity due to particle movement as viscosity drops during titration.	Applicable mainly to titrated dead oils, not to live oil systems under pressure depletion.	^70^
Gravimetric Analysis	Directly measures the mass of precipitated asphaltenes.	Offline and destructive process; not suitable for real-time monitoring.	^71^
Acoustic Resonance	Detects changes in the speed of sound as asphaltenes precipitate.	An indirect technique that requires complex interpretation and calibration.	^72^

Generally, the safe and efficient implementation of CO_2_ storage in water disposal zones is critically dependent on accurately forecasting and mitigating asphaltene deposition, a primary risk that can impair injectivity and storage integrity. However, conventional methods for assessing asphaltene behavior are often ill-suited for this specific environment. They typically require large sample volumes and involve complex, time-consuming procedures that do not adequately replicate the oil-in-water emulsion conditions prevalent near disposal wells. This gap underscores a persistent industry need for compact, efficient laboratory equipment capable of evaluating the phase behavior of crude oil in the presence of CO_2_ within emulsion systems. To address this necessity, the present study introduces an innovative technique and laboratory protocol centered on a miniature pressure-volume-temperature (PVT) cell coupled with in-situ NIR spectroscopy. This apparatus is specifically designed to monitor potential asphaltene precipitation in real-time, tracing any instability within aqueous phases containing oil droplets during CO_2_ injection. By providing a rapid and precise method to de-risk this storage strategy, this research delivers a valuable contribution to flow assurance.

## Experimental section

### Materials

The fluids used in this study consist of brine solutions (Table 2) and crude oil (Table 3). In this study, oil in water EMs were prepared using FW-crude oil, SW-crude oil, 2DSW-crude oil and 10DSW-crude oil. The high-salinity waters used in this study were synthetically prepared in the laboratory. The base SW was synthesized by dissolving a specific blend of salts into one liter of distilled water. To investigate the effect of increased salinity, two further solutions were prepared using an identical salt composition but at higher concentrations: one with 2DSW and another with 10DSW. The synthesis waters were designed to mimic the ionic composition of Persian Gulf Sea water. Additionally, a synthetic formation water, designed to mimic the ionic composition of formation water, was prepared. In all cases, after the salts were completely dissolved by stirring, the resulting brine was filtered through filter paper to remove any potential undissolved impurities. The filtrate collected after this filtration step was used as the final synthetic water for the experiments. In this study, the colloidal instability index (CII) was used to assess the stability of the crude oil. This index represents the ratio of the sum of asphaltenes and saturates to the sum of resins and aromatics. A CII value greater than 0.9 indicates an unstable crude oil^73^. Based on SARA data, the reservoir oil CII value of 2.57 suggests a high potential for asphaltene precipitation.

**Table 2:** The detailed analysis of the FW, SW, 2dSW, and 10dSW.

FW		SW		2DSW		10DSW	
Brine		Brine		Brine		Brine	
**NaCl (g.L** ^**− 1**^ **)**	154.031	**NaCl (g.L** ^**− 1**^ **)**	25.576	**NaCl (g.L** ^**− 1**^ **)**	12.788	**NaCl (g.L** ^**− 1**^ **)**	2.558
**KCl (g.L** ^**− 1**^ **)**	1.193	**KCl (g.L** ^**− 1**^ **)**	1.118	**KCl (g.L** ^**− 1**^ **)**	0.559	**KCl (g.L** ^**− 1**^ **)**	0.112
**CaCl**_**2**_ **2 H**_**2**_ **O (g.L** ^**− 1**^ **)**	24.110	**CaCl**_**2**_ **2 H**_**2**_ **O** **(g.L** ^**− 1**^ **)**	1.764	**CaCl**_**2**_ **2 H**_**2**_ **O** **(g.L** ^**− 1**^ **)**	0.882	**CaCl**_**2**_ **2 H**_**2**_ **O (g.L** ^**− 1**^ **)**	0.176
**MgCl**_**2**_ **6 H**_**2**_ **O(g.L** ^**− 1**^ **)**	8.933	**MgCl**_**2**_ **6 H**_**2**_ **O** **(g.L** ^**− 1**^ **)**	11.995	**MgCl**_**2**_ **6 H**_**2**_ **O (g.L** ^**− 1**^ **)**	5.997	**MgCl**_**2**_ **6 H**_**2**_ **O (g.L** ^**− 1**^ **)**	1.199
**Na**_**2**_**SO**_**4**_ **(g.L**^**− 1**^**)**	0.426	**Na**_**2**_ **SO**_**4**_ **(g.L** ^**− 1**^ **)**	6.818	**Na**_**2**_ **SO**_**4**_ **(g.L** ^**− 1**^ **)**	3.409	**Na**_**2**_ **SO**_**4**_ **(g.L** ^**− 1**^ **)**	0.682
**NaHCO**_**3**_ **(g.L**^**− 1**^**)**	0.672	**NaHCO**_**3**_ **(g.L**^**− 1**^**)**	0.336	**NaHCO**_**3**_ **(g.L**^**− 1**^**)**	0.168	**NaHCO**_**3**_ **(g.L**^**− 1**^**)**	0.034
**TDS (ppm)**	189,365	**TDS**	47,607	**TDS**	23,803	**TDS**	4761

**Table 3:** The physical properties of crude oil from an Iranian oil reservoir.

Physical properties	Viscosity at 80 °C	Density at 80 °C	Aromatic	Resin	Saturates	Asphaltene	Total acid number
**Crude oil (South-West Iranian Oil Field)**	7.5 (cp.)	0.84 (g/cc)	20.5 (wt %)	7.5 (wt %)	70.5 (wt %)	1.5 (wt %)	0.11 (mg KOH/g oil)

### Experimental setup

**Fig. 1:**
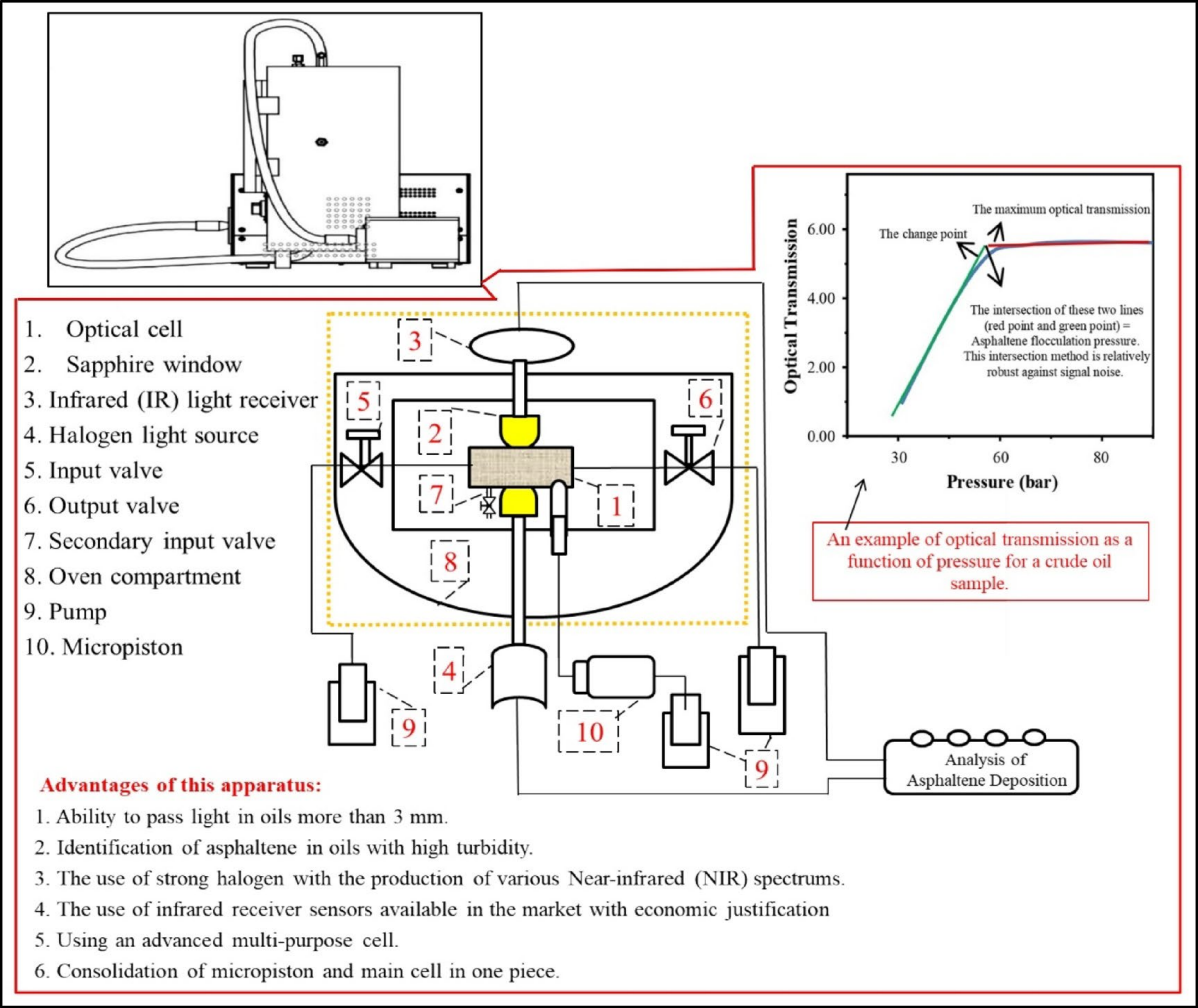
System designed for EM and asphaltene deposition/precipitation measurement.

**Fig. 2:**
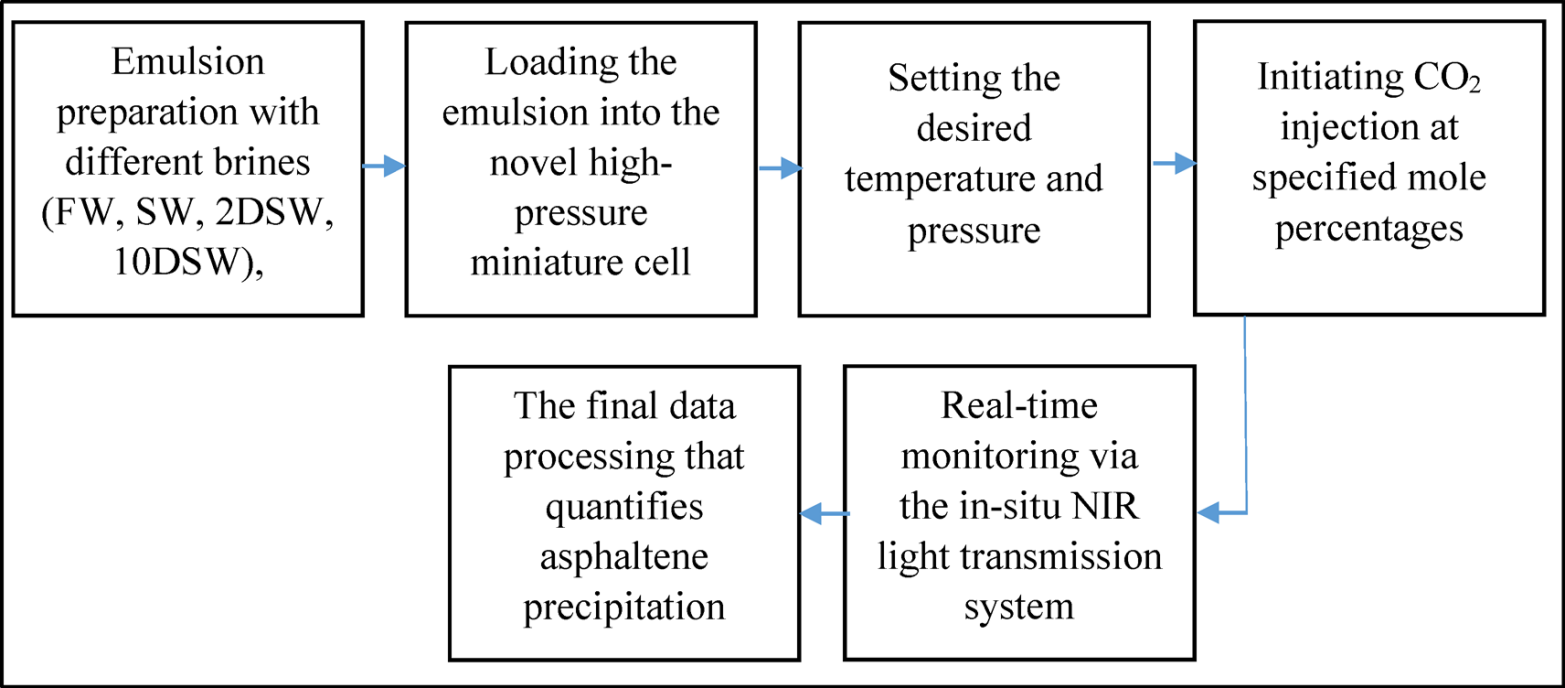
Experimental procedure flow chart.

The precise identification of asphaltene precipitation conditions in oil reservoirs remains a significant challenge. This study employs a near-infrared (NIR) technique to investigate asphaltene deposition in EM phases contacted with CO_2_. In this study, a novel miniature high-pressure PVT cell with an integrated in-situ NIR system was designed (Fig. 1) to directly monitor asphaltene instability in opaque oil-in-water emulsions under reservoir conditions. It should be noted that the technology of this innovative device was designed and manufactured at Sharif University of Technology in Iran. The apparatus features a small-volume cell (2 cc, 2 mm thickness) that enables rapid analysis of minimal sample volumes. Asphaltene precipitation is detected in real-time by a decrease in NIR light transmission, caused by particle scattering and absorption. This signal is sent to a data acquisition system which quantifies the amount of precipitated asphaltene. The system minimal dead volume ensures high analytical accuracy, while rigorous calibration underpins the reliability of the data. It is worth noting, the experimental protocol was designed with a strong emphasis on quantification and repeatability. The core measurement of asphaltene precipitation was achieved through repeated in-situ NIR light transmission. The reproducibility of the precipitation onset point and the relative change in transmission intensity was found to be within ± 5% across triplicate runs for key experimental conditions, providing high confidence in the reported trends of decreasing precipitation with temperature and higher CO_2_ injection rates. Furthermore, the operational parameters were tightly controlled; system pressure was maintained with a transducer accuracy of ± 0.1% of the full scale, and the oven temperature was controlled to within ± 1 °C of the set point. For the IFT measurements, which are crucial for explaining the behavior with 2DSW, each reported value is the average of at least five separate measurements, with a standard deviation consistently less than ± 0.2 mN/m. Generally, the flow chart of the test method is shown in Fig. 2. This integrated apparatus and methodology represent a significant technical advancement, providing a precise and efficient means of screening for asphaltene-related risks in CO_2_ storage projects.

#### Carbon dioxide

A commercial high-purity CO_2_ source (≥ 99.99%) was used for all injection experiments. The gas was supplied from a cylinder equipped with a dip tube and delivered using a high-precision syringe pump (ISCO 260D). The pump maintained a constant CO_2_ supply pressure of 500 psig and was programmed to inject a precise volume, calculated via Eqs. (1–4), to achieve the target mole fraction in the PVT cell. The entire delivery line was thermally jacketed within the oven to maintain isothermal conditions and ensure consistent CO_2_ density during injection.

### Experimental procedure

Near-Infrared (NIR) spectroscopy is a powerful analytical technique used to study the properties of materials, including the characterization of asphaltenes and their precipitation behaviors in crude oil. NIR spectroscopy involves the interaction of NIR light (typically in the wavelength range of 700 to 2500 nm) with a sample. Molecules absorb NIR light due to overtones and combination vibrations of molecular bonds (primarily C-H, N-H, O-H). The instrument measures the intensity of transmitted or reflected light at various wavelengths. The absorbed light causes transitions in molecular vibrations, leading to a spectrum that provides information about the molecular structure and concentration of specific components. On the other hand, NIR spectroscopy allows for rapid, non-destructive analysis, making it suitable for real-time monitoring of asphaltene precipitation in production environments. Continuous monitoring can help operators adjust conditions proactively to minimize precipitation issues. In this study, the asphaltene deposition is simultaneously examined by a newly developed, fast, and reliable NIR spectroscopy technique. In the novel Procedure, only less than 2 ml of sample is required for each test. Thus, in this regard, in the first stage, the EM phase consists of crude oil sample-low salinity water, namely seawater (SW), two times diluted seawater (2DSW), ten times diluted seawater (10DSW) and formation water (FW) was prepared. Therefore, a certain amount of crude oil sample was poured into a container containing FW and mixed using a mixer. Oil/Water EM (oil-in-water) phases were prepared with a ratio of 20:80. The same steps were performed for other low salinity waters (SW, 2DSW and 10DSW) with crude oil. The samples were stirred, corresponding to fluid dynamics at the wellbore region. After preparing the solutions, the oil-in-water EM solution was injected into the cell. In this regard, at first, by opening both valves connected to the tube (numbers 5 and 6) (Fig. 1), the flow lines and the tube were vacuumed. Then, the prepared EM was injected into the tube when the EM sample passed through valve 2 (number 6). In this regard, the outlet valve (number 6) was closed. Thus, the pressure of the system was increased to 20 bar using a syringe pump. Next, the inlet valve (number 2) was closed. Generally, the temperature was adjusted using the controller installed on the apparatus. At first, the light transmission was monitored without a CO_2_ injection scenario. At the end of the measurement, the asphaltene deposition of the EM was reported using the LED detector. The same procedure was performed for all emulsions ((Crude Oil/SW), (Crude Oil/2DSW), (Crude Oil/10DSW), (Crude Oil/FW)). Generally, the tests were carried out at various temperatures (30, 40, 60, 80, 100, and 120 °C). Also, in this set of tests, the light transmission for various EM samples as the CO_2_ was injected into the solution through a piston − cylinder apparatus at various mole% was measured. In general, using different temperatures and pressures of the gas cylinder available in the laboratory, the value of the z-factor was calculated using PVTsim software. On the other hand, Eqs. (1–4) was used to calculate the injected gas volume.

1 $$\:{\mathrm{Mw}}_{\mathrm{dead\:oil}}\mathrm{=\:}\sum\:{\mathrm{Z}}_{\mathrm{i}}\text{}{\mathrm{Mw}}_{\mathrm{i}\text{}}$$

2 $$\:\mathrm{2}{cc\times\:\rho}\left(\frac{\mathrm{gr}}{\mathrm{cc}}\right){\times}{\text{}\mathrm{Mw}}_{\mathrm{dead\:oil}}={\rm n_{t}}$$

3 $$\:\frac{{\mathrm{X}}_{\mathrm{C}{\mathrm{O}}_{2}}}{{\mathrm{X}}_{\mathrm{C}{\mathrm{O}}_{2}\mathrm{\:+\:}{n}_{\mathrm{t}}}}\mathrm{=}\mathrm{X}\text{}\text{}\text{}\text{}\text{}\text{}$$

4 $$\:{\mathrm{P}}_{\mathrm{cylinder}}{\:\times\:\mathrm{V}}_{\mathrm{C}{\mathrm{O}}_{\mathrm{2}}}\mathrm{=}\text{}\mathrm{Z}\text{}{times\mathrm{X}}_{\mathrm{C}{\mathrm{O}}_{\mathrm{2}}}{\times}\mathrm{R}\times\mathrm{T}$$

Here, in Eq. (1), Mw_deadoil_, Z_i_, and Mw_i_ represent the molecular weight of the dead oil, the percent composition, and the molar mass of each component, respectively. In Eqs. (2), 2 cc, ρ, and n_t_ represent the designed cell volume, density, and total number of moles. In Eq. (3), $\:{\mathrm{X}}_{\mathrm{C}{\mathrm{O}}_{2}}$, nt, and X represent the mole fraction of CO_2_, the total number of moles, and the injection amount for 20, 30, 35, 40, 45, 50% mole of CO_2_, respectively. Also, in Eq. (4), P_cylinder_,Z, $\:{\mathrm{X}}_{\mathrm{C}{\mathrm{O}}_{\mathrm{2}}}$, R, and T represent the CO_2_ cylinder pressure (psi), compressibility factor (calculated using PVTsim software), the mole fraction of CO_2_, the universal gas constant (10.731 (ft^3^.psi).(R.Ib.mol)^−1^), and temperature (°R), respectively. Therefore, by performing the calculations in Eq. (4), the $\:{\mathrm{V}}_{\mathrm{C}{\mathrm{O}}_{\mathrm{2}}}$ is calculated.

### Interfacial tension measurement

To investigate the fluid-fluid interactions between oil and brine, the pendant drop technique was employed to measure interfacial tension. The pendant drop method is a well-established technique for measuring interfacial tension under environmental conditions. To perform the measurement, a U-shaped syringe is utilized to position an oil droplet at its tip. Initially, a chamber that allows for light exposure is filled with low-salinity water. Once the low-salinity water is prepared, the syringe tip, containing the oil droplet, is placed above the chamber. A camera is then used to capture an image of the droplet. Subsequently, the software analyzes the recorded image to determine the interfacial tension. Generally, the bulk densities of the oil phase and the gas phase (pure CO_2_ or thickened CO_2_) required for the IFT calculations using the pendant drop technique were determined through direct measurement and established software, respectively. For the oil phase, the density was measured directly at reservoir conditions using DMA HPM Anton Paar apparatus^74^. For the gas phase, the density of pure CO_2_ was obtained using the PPDS software, which is a recognized database for fluid properties. The PPDS software is used for measuring the density of pure CO_2_, which retains a database for fluid properties developed by the National Engineering Laboratory in the UK^75–77^.

### Test design

#### Identification of effective parameters

Considering the shallow water disposal formation at the mentioned oil field, the pressure was maintained constant at 20 bar. Since the temperature of the injected water is affected by seasons, a wide range of temperatures was considered for the test design. Besides, the amount of injected gas would be critical to any interaction between the EM samples and the injected CO_2_. Therefore, the second parameter was set to be the CO_2_ mole%. The effect of water salinity on the EM phase was also investigated here. In the first step, 20 cc of oil was slowly poured into a beaker containing 80 cc of brine with specific salinity ranging from FW to 10DSW. The samples were stirred by a Vortex Mixer at 6000 rpm for 45 min. After 45 min of the emulsification (oil in water) process, several small samples were collected to evaluate CO_2_ storage in oil reservoir water disposal zones and asphaltene deposition monitoring. The tests to find the temperature effect on asphaltene precipitation were designed to cover the range of temperatures from 30 to 120 °C. After the EM samples (oil in water) were prepared, the process of injecting those samples into the cell in the apparatus was performed. Besides, different mole% of injecting CO_2_ was tested, as the results are presented in the next section.

#### Test procedure

As it was explained earlier, two main parameters, namely temperature and CO_2_ mole%, were set to be tested in this experimental work. Thus, after the EM (oil in water) preparation process (According to Sect. "Identification of effective parameters"), the process of injecting salinity types and formation water into the cell in the apparatus was done (at different CO_2_ (mol%), pressure (20 bar) and temperature (30°, 40°, 60°, 80°, 100°, 120 °C). Generally, all tests were performed under pressure (20 bar) and at different temperatures and different gas injection rates. Each test was repeated at least three times to ensure reproducibility of results. The test design followed in this experimental work is comprehensively reported in Table 4. The tests are performed based on the salinity and the type of ions dissolved in water, system temperature, pressure and CO_2_ rate (mol%).

**Table 4:** Experiment design to conduct experiments using the newly developed innovative device.

Tests Category	Test#	Water type	T (°C)	CO_2_ rate (mol%)	*P* (bar)
Category 1	1–4	SW, 2DSW, 10DSW, FW	30	Without CO_2_ Injection	20
Category 2	5–8	SW, 2DSW, 10DSW, FW	40	Without CO_2_ Injection	20
Category 3	9–12	SW, 2DSW, 10DSW, FW	60	Without CO_2_ Injection	20
Category 4	13–16	SW, 2DSW, 10DSW, FW	80	Without CO_2_ Injection	20
Category 5	17–20	SW, 2DSW, 10DSW, FW	100	Without CO_2_ Injection	20
Category 6	21–24	SW, 2DSW, 10DSW, FW	120	Without CO_2_ Injection	20
Category 7	25–28	SW, 2DSW, 10DSW, FW	30	20	20
Category 8	29–32	SW, 2DSW, 10DSW, FW	30	30	20
Category 9	33–36	SW, 2DSW, 10DSW, FW	30	35	20
Category 10	37–40	SW, 2DSW, 10DSW, FW	30	40	20
Category 11	41–44	SW, 2DSW, 10DSW, FW	30	45	20
Category 12	45–48	SW, 2DSW, 10DSW, FW	30	50	20
Category 13	49–52	SW, 2DSW, 10DSW, FW	40	20	20
Category 14	53–56	SW, 2DSW, 10DSW, FW	40	30	20
Category 15	57–60	SW, 2DSW, 10DSW, FW	40	35	20
Category 16	611 − 64	SW, 2DSW, 10DSW, FW	40	40	20
Category 17	65–68	SW, 2DSW, 10DSW, FW	40	45	20
Category 18	69–72	SW, 2DSW, 10DSW, FW	40	50	20
Category 19	73–76	SW, 2DSW, 10DSW, FW	60	20	20
Category 20	77–80	SW, 2DSW, 10DSW, FW	60	30	20
Category 21	81–84	SW, 2DSW, 10DSW, FW	60	35	20
Category 22	85–88	SW, 2DSW, 10DSW, FW	60	40	20
Category 23	89–92	SW, 2DSW, 10DSW, FW	60	45	20
Category 24	93–96	SW, 2DSW, 10DSW, FW	60	50	20
Category 25	97–100	SW, 2DSW, 10DSW, FW	80	20	20
Category 26	101–104	SW, 2DSW, 10DSW, FW	80	30	20
Category 27	105–108	SW, 2DSW, 10DSW, FW	80	35	20
Category 28	109–112	SW, 2DSW, 10DSW, FW	80	40	20
Category 29	113–116	SW, 2DSW, 10DSW, FW	80	45	20
Category 30	117–120	SW, 2DSW, 10DSW, FW	80	50	20
Category 31	121–124	SW, 2DSW, 10DSW, FW	100	20	20
Category 32	125–128	SW, 2DSW, 10DSW, FW	100	30	20
Category 33	129–1332	SW, 2DSW, 10DSW, FW	100	35	20
Category 34	133–136	SW, 2DSW, 10DSW, FW	100	40	20
Category 35	137–140	SW, 2DSW, 10DSW, FW	100	45	20
Category 36	141–144	SW, 2DSW, 10DSW, FW	100	50	20
Category 37	145–148	SW, 2DSW, 10DSW, FW	120	20	20
Category 38	149–152	SW, 2DSW, 10DSW, FW	120	30	20
Category 39	153–156	SW, 2DSW, 10DSW, FW	120	35	20
Category 40	157–160	SW, 2DSW, 10DSW, FW	120	40	20
Category 41	161–164	SW, 2DSW, 10DSW, FW	120	45	20
Category 42	165–168	SW, 2DSW, 10DSW, FW	120	50	20

## Results and discussion

The impact of CO_2_ injection into water disposal wells from the oil reservoirs under enhanced oil recovery operation, using low salinity water injection, which contains EM phase, was studied. Asphaltene precipitation was measured by preparing 168 samples (42 categories) of EM phases without the presence of carbon dioxide and in the presence of carbon dioxide for different carbon dioxide mol% at a pressure of 20 bar and various temperatures (20, 40, 60, 80, 100, and 120 °C).

### The effects of different temperature scenarios on the amount of asphaltene in the emulsion

**Fig. 3:**
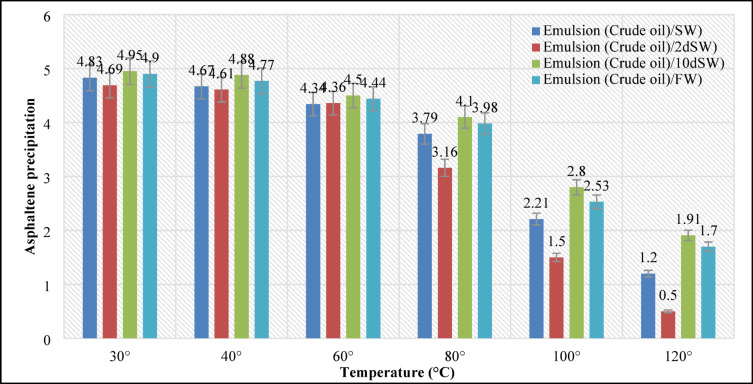
Asphaltene precipitation at different temperatures in the oil-water emulsion.

In this section, 24 types (categories 1–6 in Table 4) of EM samples were prepared using 20 volume% crude oil and 80 volume% of different brine samples. In this regard, the results of asphaltene precipitation in the emulsions of different salinities for the range of temperature change (20–120 °C) are shown in Fig. 3. As shown in Fig. 3, the extent of asphaltene precipitation, quantified by the reduction in NIR transmission, decreased consistently with increasing temperature across all brine salinities. Temperature would highly affect the CO_2_ dissolution in the brine samples as it almost decreases to 10% as the sample is heated from 20 °C – 120°C^30^.

### The effects of CO_2_ concentration and interfacial tension investigation

**Fig. 4:**
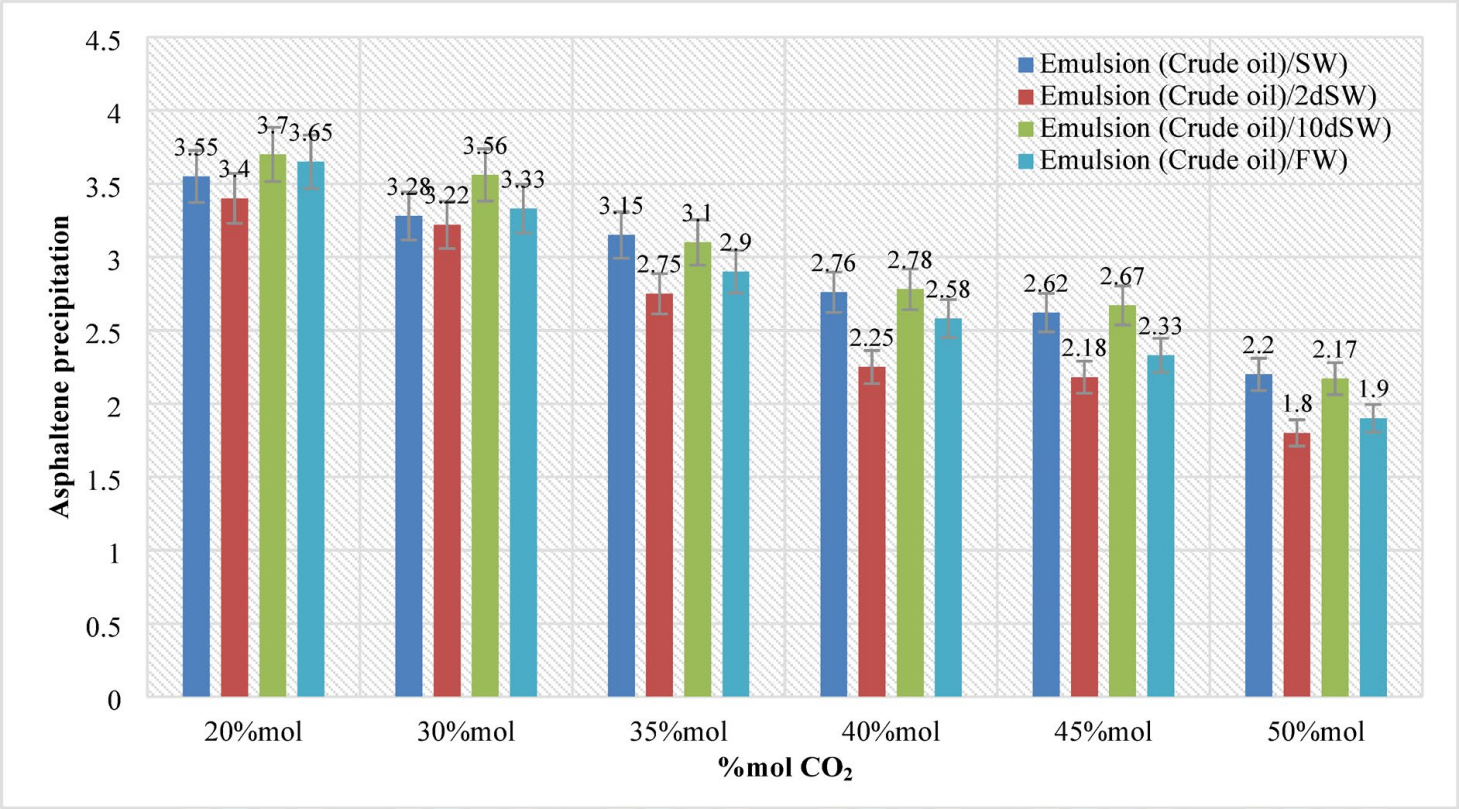
Asphaltene precipitation during different CO_2_ injection at pressure (20 bar) and temperature (30 °C).

**Fig. 5:**
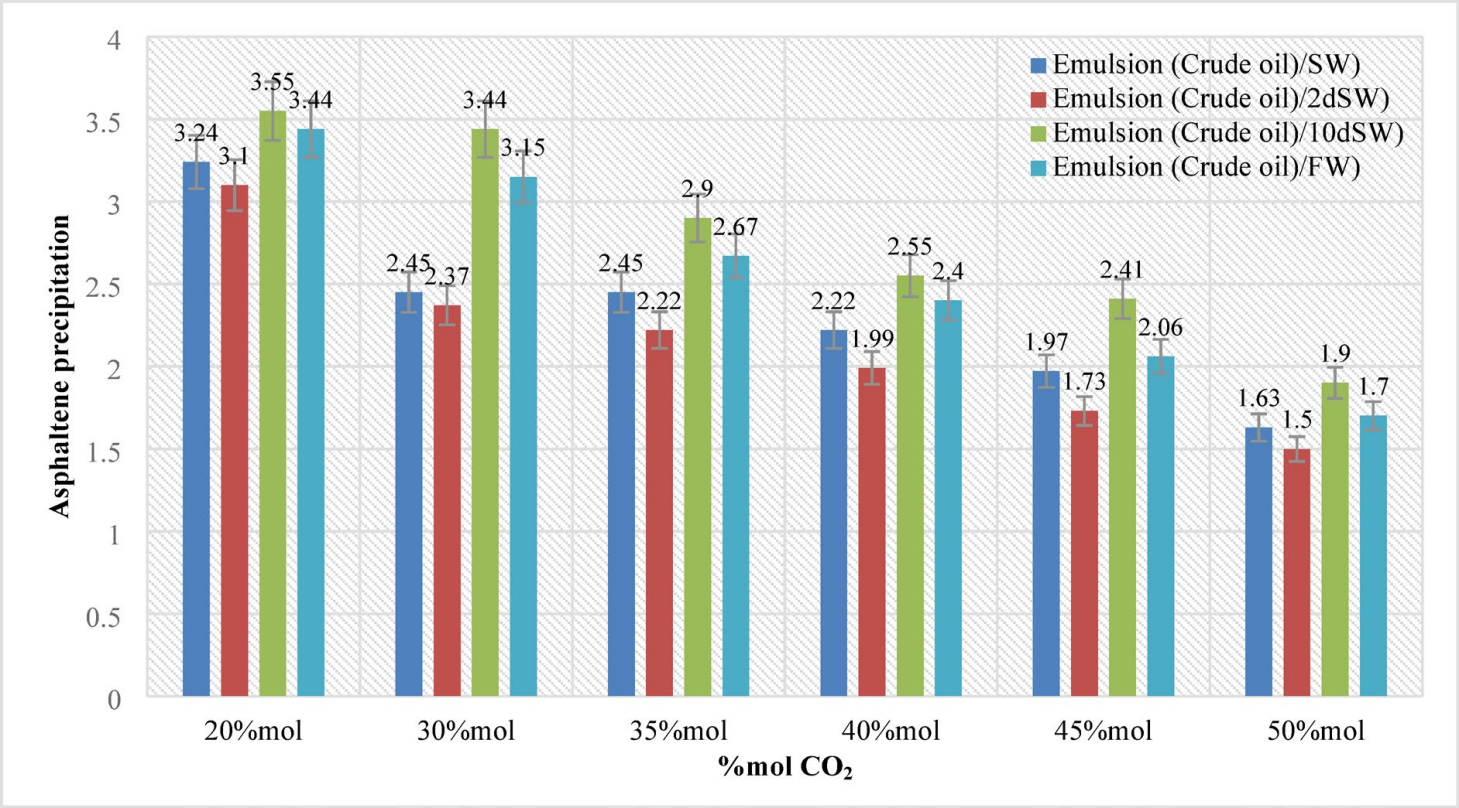
Asphaltene precipitation during different CO_2_ injection at pressure (20 bar) and temperature (40 °C).

**Fig. 6:**
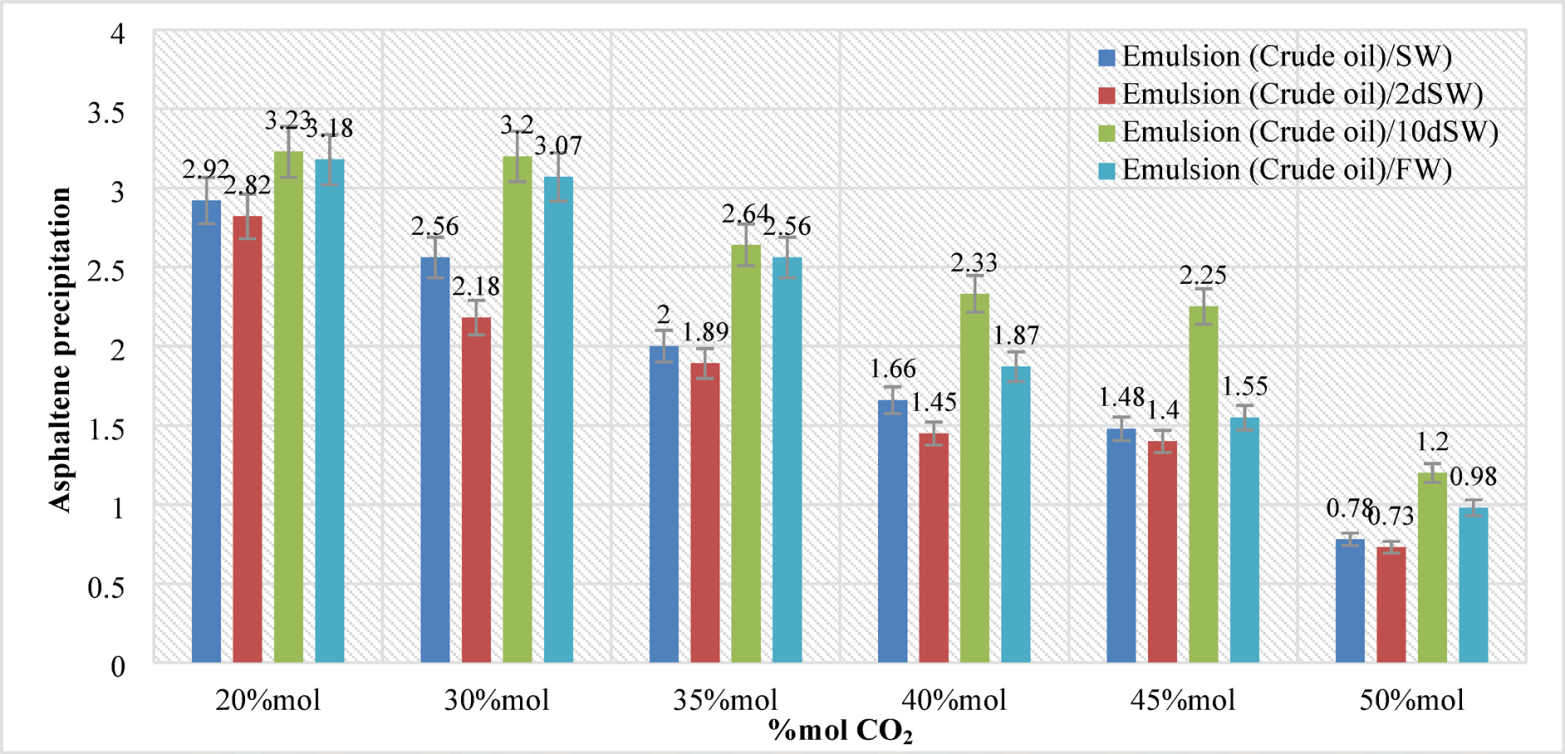
Asphaltene precipitation during different CO_2_ injection at pressure (20 bar) and temperature (60 °C).

**Fig. 7:**
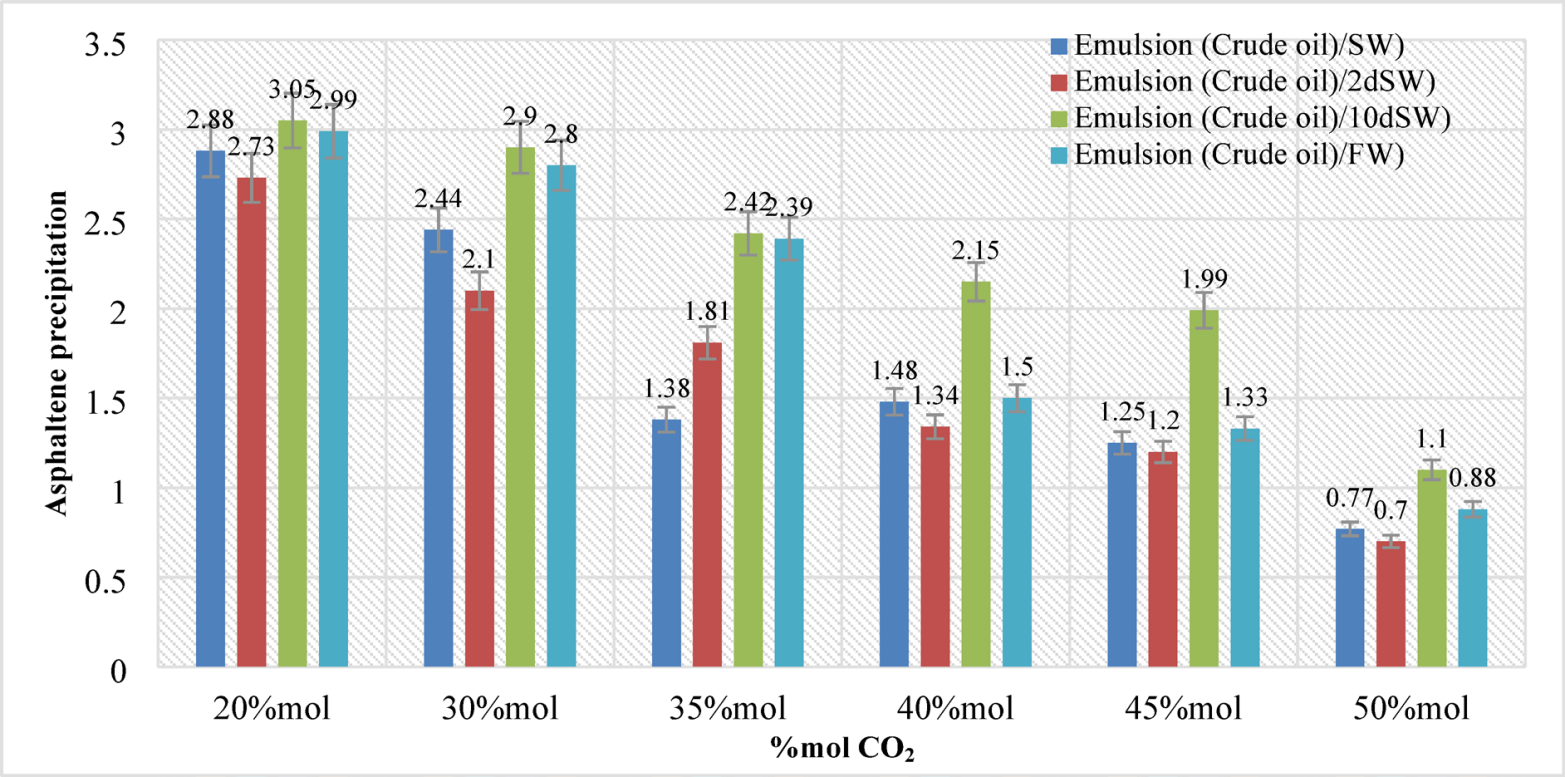
Asphaltene precipitation during different CO_2_ injection at pressure (20 bar) and temperature (80 °C).

**Fig. 8:**
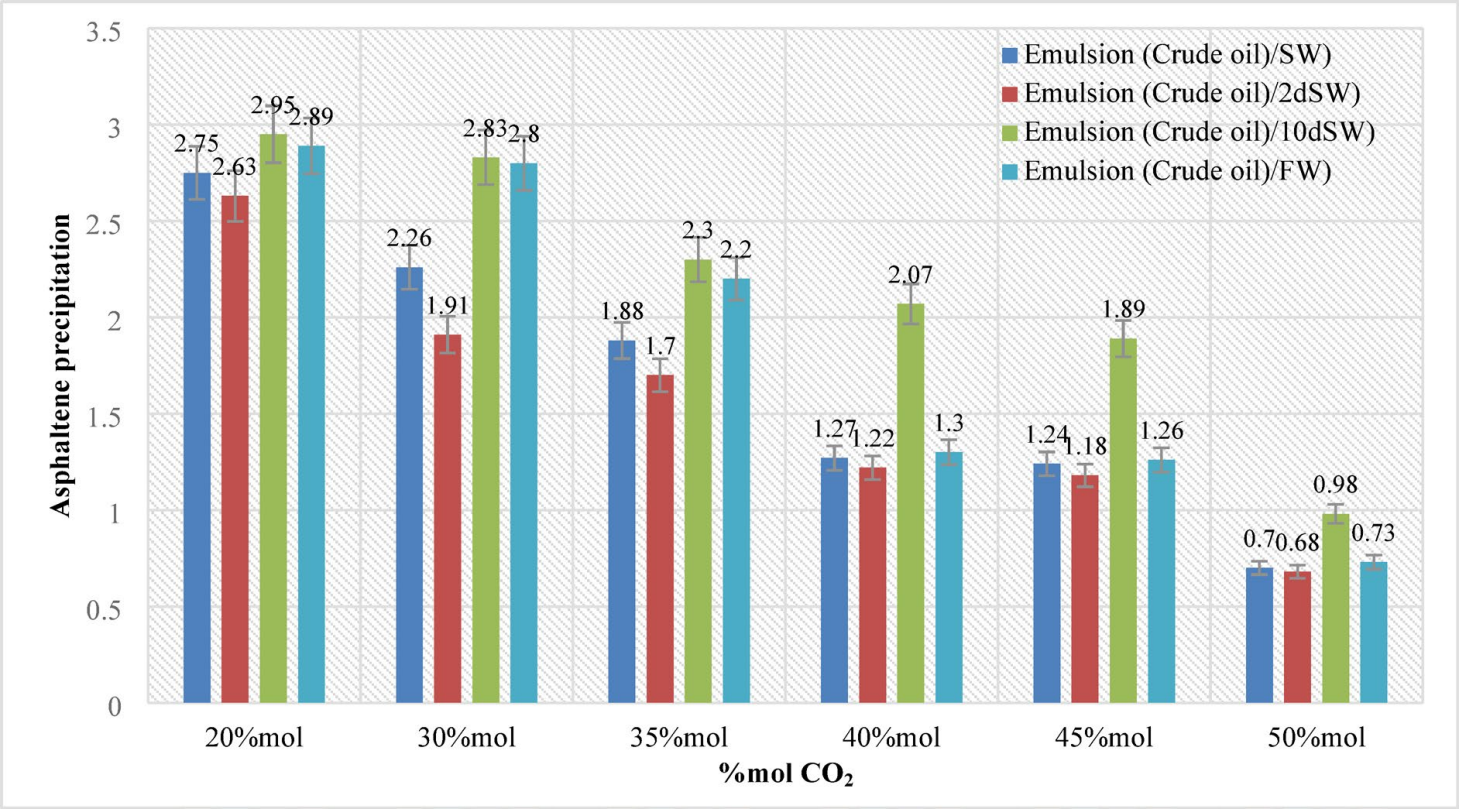
Asphaltene precipitation during different CO_2_ injection at pressure (20 bar) and temperature (100 °C).

**Fig. 9:**
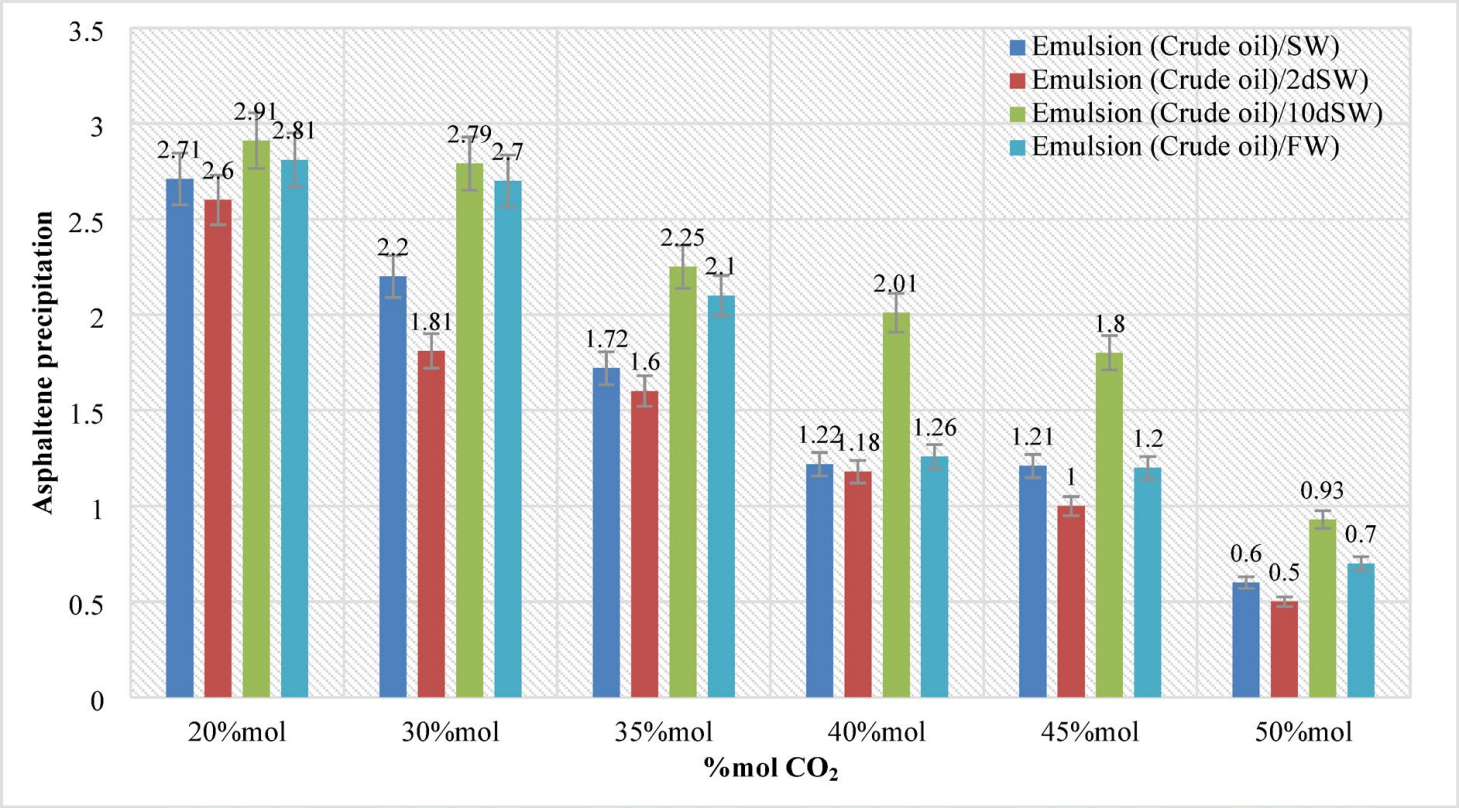
Asphaltene precipitation during CO_2_ injection at pressure (20 bar) and temperature (120 °C).

**Fig. 10:**
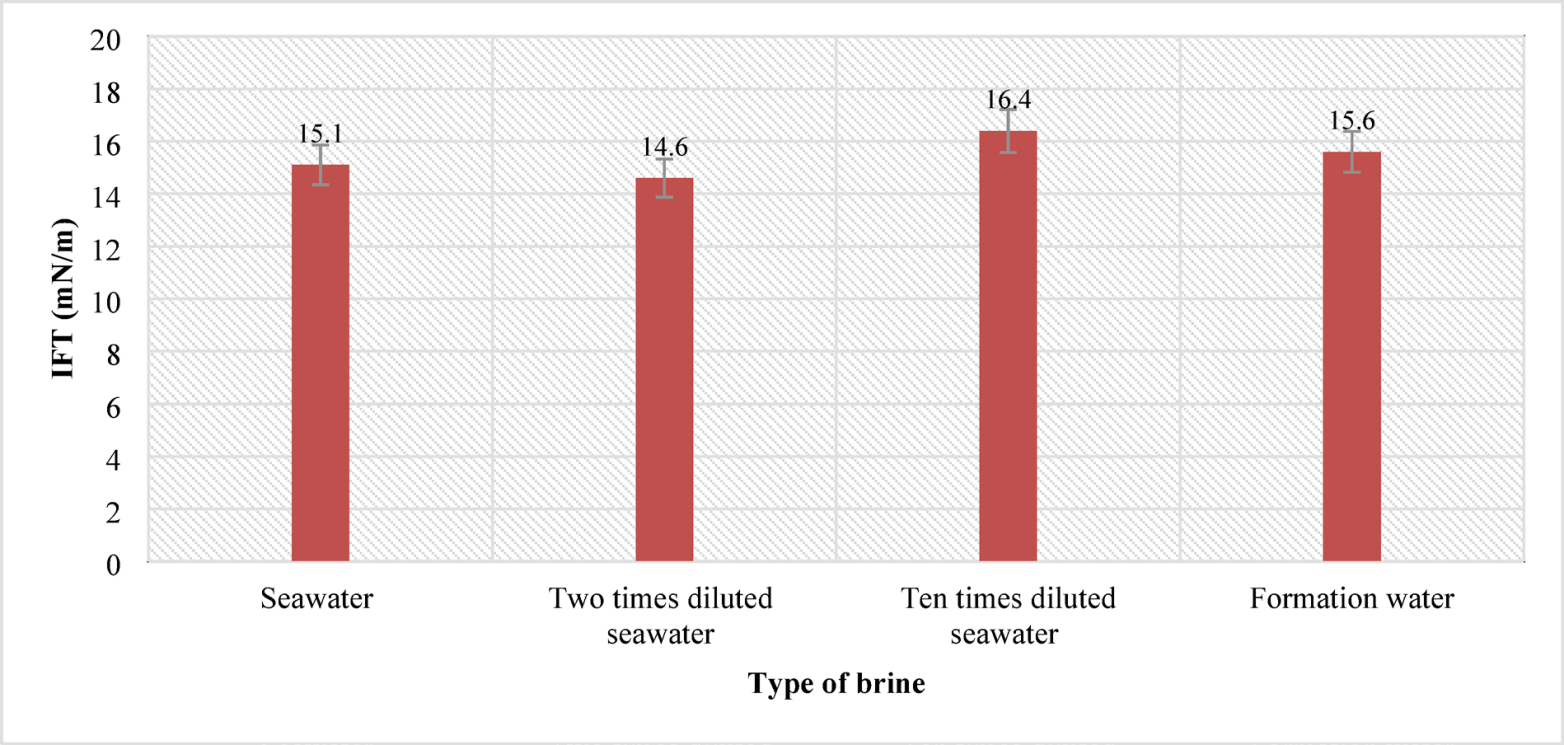
Interfacial tension between brines and oil sample.

**Fig. 11:**
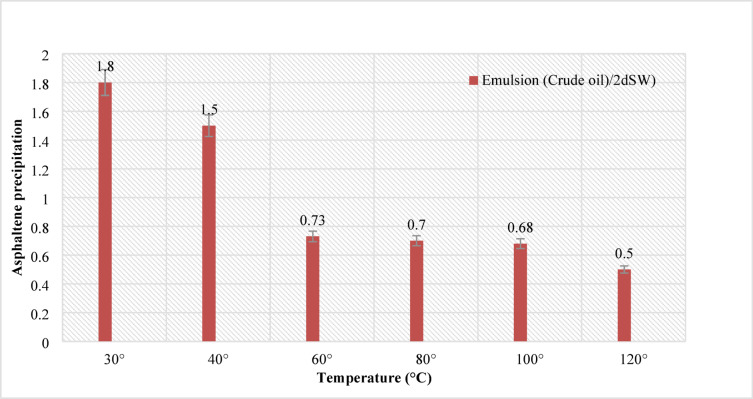
Asphaltene precipitation in CO_2_ injection (50 mol%), pressure (20 bar) and different temperatures for emulsion (Crude Oil/2DSW).

In this section, 144 samples (categories 7–42 in Table 4) of oil-in-water EM samples were prepared. The results indicate that a higher amount of injected CO_2_ would cause less asphaltene precipitation (Table 5). This phenomenon is also critically affected by temperature changes as it was already described in the previous section. As for the amount of CO_2_ injection (based on mole%), the results presented in Figs. (4–9) indicated lower asphaltene precipitation at higher CO_2_ injection. This phenomenon is explained based on the limited CO_2_ dissolution in the aqueous phase at the test conditions and the low amount of crude oil in the EM phase (20 volume %). Furthermore, at high CO_2_ injection rates (above 35 mol%), the crude oil in the EM phase is likely subject to vaporization, resulting in fewer liquid crude oil droplets available to interact with the dissolved CO_2_ gas. These results are well described in Figs. (4–9) for the case of CO_2_ injection of 35 to 50 mol%, more CO_2_ available leads to less volume of crude oil accessible to the carbon dioxide dissolved in the EM phase. This theory is well supported by comparing the results for high CO_2_ mole% at elevated temperature, as they are described in Figs. (4–9). The other parameter that are presented here is the salinity of the brine samples used for the EM phase preparation. The results presented here indicate a lower amount of asphaltene precipitation for the case of 2DSW. This is well confirmed by the results presented by our experimental work shows that minimum interfacial tension is achieved for the 2DSW case (Fig. 10). However, there is confidence in the importance of this trend for two main reasons, one statistical and the other mechanical. Firstly, while the IFT values are in a relatively narrow range, the data presented in Fig. 10 is the result of highly reproducible measurements. The observed trend—where 2DSW consistently yields the lowest IFT, compared to standard SW was statistically significant across all experimental replicates. Although in several reports it is shown that the IFT of crude oil and 10DSW is marginally lower than 2DW, however in many cases it is preferred to use 2DW because of cost saving of desalination process. It is worth noting that the mechanisms have been critically reviewed in two published works by our research group^55,78^. On the other hand, this means a lower size for oil droplets that would facilitate more crude oil to be evaporated at a higher amount of CO_2_ available. This fact is almost visible in all cases described in this research work (Table 5). The previous studies indicated that a lower IFT corresponds to a more stable emulsion^78^. We suggest that these smaller droplets, created by the low IFT of 2DSW, are more susceptible to the phase-behavior change induced by high CO_2_ concentrations. The larger surface area facilitates faster and more extensive mass transfer, allowing the system to cross the thermodynamic threshold into a regime where the crude oil components are effectively vaporized or drastically altered, leaving fewer asphaltenes available to precipitate. In general, as temperature increases (based on experimental results), less asphaltene precipitation is observed. This is related to the amount of CO_2_ dissolving in both the water and oil phases at an elevated temperature, which hinders the lower chance for asphaltene precipitation. The general results of these tests would confirm lower asphaltene precipitation challenges for the cases of 2DSW aqueous phase, higher amount of CO_2_ injection rate and high-temperature conditions. In this regard, Fig. 11 describe the summary of the results for different temperatures and CO_2_ injection rates that support the explanation in this section. These results show that even very small amount of asphaltene precipitation is detected by the NIR device. Generally, for instance, at 30 °C and 35 mol% CO_2_, using 2DSW reduces asphaltene precipitation by 54% compared to FW. At 50 mol% CO_2_, the reduction is 73%. This demonstrates that 2DSW is the most effective single mitigation strategy. Compare high vs. medium injection rates. For example, increasing CO_2_ from 35 mol% to 50 mol% reduces precipitation by 39% for FW at 30 °C and by 63% for 2DSW at 30 °C, supporting the theory of a phase-change “vaporization regime.” The optimal condition (2DSW, 120 °C, 50 mol% CO_2_) results in a 97% reduction in precipitation compared to the worst-case (FW, 30 °C, 35 mol% CO_2_). This shows the powerful synergy of combining all optimal parameters. In this study, a complete quantitative data set of NIR measurements was available for all 168 scenarios. In this regard, the NIR system was calibrated to convert the reduction of light transmission (due to asphaltene scattering and absorption) into a relative measure of asphaltene precipitation. The core measurement was the Relative Change in NIR Transmission Intensity (ΔT/T₀), where any reduction indicates asphaltene precipitation. This metric is directly proportional to the amount of asphaltene particles obstructing the light path. For context, the baseline transmission (T₀) for a particle-free, stable emulsion was set at 100% for each test series. In this regard, Table 5 summarizes the quantitative data for the most significant conditions discussed in the manuscript, demonstrating the key trends. In this study, the main mechanisms are dissolution of CO_2_ in water and trapping in the large pore.

**Table 5:** Quantitative summary of asphaltene precipitation (Reported as % Reduction in NIR Transmission).

Water Type	Temperature (°C)	CO_2_ (mol%)	Avg. % Reduction in Transmission (± Std. Dev.)	Key Trend
FW	30	0	8.5% (± 0.7)	Baseline without CO_2_
2DSW	30	0	2.1% (± 0.3)	2DSW shows least precipitation
FW	30	20	22.3% (± 1.2)	Classic destabilization
2DSW	30	20	9.8% (± 0.9)	2DSW mitigates effect
FW	30	35	25.1% (± 1.5)	Peak precipitation
2DSW	30	35	11.5% (± 1.1)	-
FW	30	50	15.4% (± 1.8)	Reduction at high CO_2_
2DSW	30	50	4.2% (± 0.5)	Minimal precipitation
2DSW	60	50	2.5% (± 0.4)	Combined effect of T and CO_2_
2DSW	120	50	0.8% (± 0.2)	Optimal Condition

### CO_2_ storage in water disposal wells and the balance between environmental benefits and asphaltene risks

As the world seeks scalable solutions to combat climate change, an emerging strategy combines carbon sequestration with produced water management by repurposing disposal wells—traditionally used for injecting waste fluids from oil and gas operations—into storage sites for captured CO_2_. This approach not only reduces greenhouse gas emissions by securely storing CO_2_ underground but also optimizes existing infrastructure, cutting costs associated with drilling new wells. When injected into water-filled disposal wells, CO_2_ either dissolves or forms a separate phase, depending on subsurface conditions, though careful pressure management is crucial to prevent leakage or reservoir damage. Beyond emissions reduction, this method addresses the environmental challenge of produced water disposal, as reinjecting CO_2_ can offset the volume of wastewater while stabilizing underground formations. However, key technical risks, such as asphaltene precipitation—where CO_2_ interacts with residual oil, potentially clogging pores and reducing permeability—must be mitigated through advanced monitoring and chemical treatments. Despite these challenges, the deep geological layers used for storage are typically isolated from freshwater aquifers by impermeable rock, minimizing contamination risks, while the injected CO_2_ can help maintain reservoir pressure, further protecting groundwater. By integrating CO_2_ storage with produced water management, this dual-purpose strategy enhances both economic and environmental efficiency, offering a circular solution that aligns industrial operations with climate goals. Ultimately, this innovative use of disposal wells exemplifies how repurposing existing infrastructure can drive sustainable progress, turning waste management into a climate solution and advancing the transition toward a low-carbon future.

### Future directions

Generally, this study offer valuable insights for optimizing by modifying operational factors like waterflooding rates and pressure maintenance to reduce asphaltene deposition and precipitation. Moreover, recognizing the role of 2DSW as a stabilizing agent opens up novel possibilities for improving EM management. These results highlight the necessity of adapting techniques to particular reservoir states, such as temperature, pressure, oil-water composition, and water content, for CO_2_ storage in water disposal zones of oil reservoirs and monitoring asphaltene deposition. Future directions must concentrate on the long-term stability and behavior of EMs under a wider variety of reservoir states. It should also include simulation studies examining how ions in 2DSW interact with asphaltenes to better understand precipitation and EM stabilization processes.

## Conclusions

While oil-water emulsions (EMs) have long been a characteristic feature of oil production, their role in carbon sequestration introduces new challenges. This study establishes an experimental protocol to detect asphaltene precipitation challenges during CO_2_ storage in water disposal zones. The key to engineering application lies in selecting the optimized conditions identified here: using a 2DSW emulsion at 120 °C, 20 bar, and a high CO_2_ injection concentration (50 mol%). Under these conditions, asphaltene precipitation—measured as the reduction in NIR transmission—was minimized to a mere 0.8% (± 0.2). This represents a substantial quantitative improvement compared to the baseline scenario using FW at 30 °C without CO_2_, which showed an 8.5% (± 0.7) reduction, or even the worst-case scenario of FW with 35 mol% CO_2_ at 30 °C, which peaked at a 25.1% (± 1.5) reduction. This quantitative data translates into direct engineering value. First, it provides a clear screening criterion: aqueous phase salinity is not merely an influential factor, but the use of 2DSW is a specific mitigation strategy that can reduce asphaltene precipitation by over 80% compared to high-salinity formation water under challenging conditions. Second, limiting CO_2_ injection to moderate levels minimizes asphaltene precipitation, as our results demonstrate that higher concentrations (> 35 mol%) can induce a phase-behavior crossover into a “vaporization” regime, which proactively prevents the associated formation damage. Furthermore, the conclusion emphasizes that our developed miniature PVT cell with integrated NIR spectroscopy is not just a novel apparatus but a practical tool for rapid, small-volume screening of parameters related to asphaltene precipitation challenges. The high reproducibility of the protocol ensures it can reliably generate site-specific data, enabling operators to tailor injection parameters—such as brine composition, temperature, and CO_2_ injection rate—to their specific reservoir fluids. Essentially, this work provides a quantitative framework and a specialized toolset for designing CO_2_ storage projects in water-disposal zones. These projects maximize injectivity and storage integrity by minimizing asphaltene-related formation damage to negligible levels, thereby directly reducing risks in project planning and execution. The practical significance of this research lies in providing a robust methodology to mitigate risks associated with CO_2_ injection into water-saturated zones, including disposal wells and water-flooded reservoirs. By enabling accurate prediction of asphaltene deposition within representative oil-in-water EMs, our results allow operators to proactively prevent wellbore impairment and formation damage.

## Data Availability

All data generated or analysed during this study are included in this article.

## Abbreviations

10DSW: Ten times diluted seawater
2DSW: Two times diluted seawater
EM: Emulsion
FW: Formation water
SW: Seawater

## References

[1] Abas, N., Kalair, A., Khan, N. & Kalair, A. R. Review of GHG emissions in Pakistan compared to SAARC countries. *Renew. Sustain. Energy Rev.* **80**, 990–1016 (2017).

[2] Jeffry, L. et al. Greenhouse gases utilization: a review. *Fuel* **301**, 121017 (2021).

[3] European Commission (EC). Towards an integrated strategic energy technology (SET) plan: research and innovation challenges and needs of the EU energy system. *Communication European. Commission*. https://setis.ec.europa.eu/set-plan-process/integrated-roadmap-and-action-plan. (2013).

[4] European Commission (EC). Towards an integrated strategic energy technology (SET) plan: accelerating the European energy system transformation. *Communication European Commission*. https://ec.europa.eu/energy/sites/ener/files/documents/1_EN_ACT_part1_v8_0.pd. (2015).

[5] Hanson, E., Nwakile, C. & Hammed, V. O. Carbon capture, utilization, and storage (CCUS) technologies: evaluating the effectiveness of advanced CCUS solutions for reducing CO_2_ emissions. *Results Surf. Interfaces*. **18**, 100381 (2024).

[6] Halari, D. et al. Enhanced oil recovery using carbonated polymeric nanofluids: A new age approach to CO_2_ utilization and corrosion mitigation. *Ind. Eng. Chem.* **149**, 801–817 (2025).

[7] Bastami, D., Shahrabadi, A., Naderi, H., Taghikhani, V. & Taheri-Shakib, J. Molecular level characterization of interactions between asphaltene and solid surface for forecasting changes in wettability. *Sci. Rep.* **15**, 28394 (2025). 40759995 10.1038/s41598-025-12599-y PMC12322175

[8] Salimi, F., Ayatollahi, S. & Vafaei-Seftie, M. Prediction of asphaltene deposition during turbulent flow using heat transfer approach. *Pet. Sci. Technol.* **36**, 632–639 (2018).

[9] Ameli, F., Hemmati-Sarapardeh, A., Dabir, B. & Mohammadi, A. H. On the evaluation of asphaltene precipitation Titration data: modeling and data assessment. *Fluid Phase Equilib.* **412**, 235–248 (2016).

[10] Hemmati-Sarapardeh, A., Dabir, B., Ahmadi, M., Mohammadi, A. H. & Husein, M. M. Toward mechanistic Understanding of asphaltene aggregation behavior in toluene: the roles of asphaltene structure, aging time, temperature, and ultrasonic radiation. *J. Mol. Liq*. **264**, 410–424 (2018).

[11] Bazyar, S., Nabipour, M., Azdarpour, A., Honarvar, B. & Esfandiari, N. Experimental investigation of asphaltene precipitation during waterflooding using different aqueous phases. *Energy Sources Part. A*. **45**, 4481–4500 (2023).

[12] Dehghani, F., Ayatollahi, S., Jafarbeigi, E. & Moradpour, N. Experimental study of asphaltene onset condition and deposition using electrical deposition technique in the presence of various additives: A novel strategy. *Fuel* **357**, 129514 (2024).

[13] Jafarbeigi, E., Sahraei, E. & Maroufi, K. A novel functionalized nanoparticle for inhibiting asphaltene precipitation and deposition. *Phys. Fluids*. **37**, 017164 (2025).

[14] Salehzadeh, M., Husein, M. M., Ghotbi, C., Dabir, B. & Taghikhani, V. In-depth characterization of light, medium and heavy oil asphaltenes as well as asphaltenes subfractions. *Fuel* **324**, 124525 (2022).

[15] Shadman, M. M., Badizad, M. H., Dehghanizadeh, M. & Dehaghani, A. H. S. Developing a novel colloidal model for predicting asphaltene precipitation from crude oil by alkane Dilution. *J. Mol. Liq*. **318**, 113879 (2020).

[16] Mahmoudvand, M., Javadi, A. & Pourafshary, P. Brine ions impacts on water-oil dynamic interfacial properties considering asphaltene and maltene constituents. *Colloids Surf. A*. **579**, 123665 (2019).

[17] Eskini, F., Dehaghani, A. S. & Shadman, M. M. Modelling the effect of the inhibitors on asphaltene precipitation using Flory–Huggins theory. *Sci. Rep.* **12** (1), 18946 (2022). 36347921 10.1038/s41598-022-23596-w PMC9643540

[18] Razghandi, M. et al. Effect of asphaltene flocculation size on deposition using a modified quartz crystal microbalance in-house setup and microscopic image analysis: model oil, inhibitory and nanoparticle solutions case studies. *Geoenergy Sci. Eng.* **237**, 212819 (2024).

[19] Taha, S. M. & Khaksar-Manshad, A. State of asphaltene in crude oil and application of nano-chemicals for aggregation inhibition: A comprehensive review. *Fuel* **393**, 135004 (2025).

[20] Amiri-Ramsheh, B., Sahebalzamani, S., Zabihi, R. & Hemmati-Sarapardeh, A. Predicting asphaltene precipitation during natural depletion of oil reservoirs by integrating SARA fractions with advanced intelligent models. *Sci. Rep.* **15** (1), 26214 (2025). 40683969 10.1038/s41598-025-11966-z PMC12276298

[21] Khalighi, J. & Cheremisin, A. Robust asphaltene onset pressure prediction using ensemble learning. *Results Eng.* **24**, 103483 (2024).

[22] Nguyen, M. T. T. et al. Molecular mechanisms of asphaltene dispersion by acrylamide-based polymers: A combined study of molecular dynamics simulations, geometric analysis, and density functional theory calculations. *J. Mol. Liq*. **414**, 132215 (2025).

[23] Hernández, E. A., Lira-Galeana, C. & Ancheyta, J. Analysis of asphaltene precipitation models from solubility and Thermodynamic-Colloidal theories. *Processes* **11**, 765 (2023).

[24] Martyushev, D. A. Modeling and prediction of asphaltene-resin-paraffinic substances deposits in oil production wells. *Georesursy* **22**, 86–92 (2020).

[25] Lashkarbolooki, M., Riazi, M., Ayatollahi, S. & Hezave, A. Z. Synergy effects of ions, resin, and asphaltene on interfacial tension of acidic crude oil and low–high salinity Brines. *Fuel* **165**, 75–85 (2016).

[26] Khormali, A., Sharifov, A. R. & Torba, D. I. The control of asphaltene precipitation in oil wells. *Pet. Sci. Technol.* **36**, 443–449 (2018).

[27] Nikoo, A. H., Ghaedi, M., Malayeri, M. R. & Riazi, M. Analysis of wellbore clogging by asphaltene deposition using interaction energies. *Fuel* **352**, 129111 (2023).

[28] Nikoo, A. H., Ghaedi, M. & Malayeri, M. R. Impact of various aggregation kinetics on thermophoretic velocity of asphaltene deposition. *Sci. Rep.* **14**, 18430 (2024). 39117792 10.1038/s41598-024-69503-3 PMC11310216

[29] Zanganeh, P., Dashti, H. & Ayatollahi, S. Comparing the effects of CH_4_, CO_2_, and N_2_ injection on asphaltene precipitation and deposition at reservoir condition: A visual and modeling study. *Fuel* **217**, 633–641 (2018).

[30] Ahmadi, Y., Kharrat, R., Hashemi, A., Bahrami, P. & Mahdavi, S. Effect of temperature on asphaltene precipitation in crude oils from Xinjiang oilfield. *ACS Omega*. **7**, 36244–36253 (2022). 36278113 10.1021/acsomega.2c03630 PMC9583088

[31] Ahmadi, Y., Akbari, A., Mansouri, M., Alibak, A. H. & Vaferi, B. Innovative Xanthan gum-based nanocomposites for asphaltene precipitation prevention in shale and carbonate rocks. *Int. J. Biol. Macromol.* **280**, 136331 (2024). 10.1016/j.ijbiomac.2024.136331 39482134

[32] Ali, S. I. et al. Phenomena, factors of wax deposition and its management strategies. *Arab. J. Geosci.* **15**, 133 (2023).

[33] Ali, S. I. et al. Factorial analysis of experimental parameters effecting asphaltene precipitation in dead crude oils. *Arab. J. Sci. Eng.* **48**, 9519–9533 (2023).

[34] Martins, R. G., Martins, L. S. & Santos, R. G. Effects of Short-Chain n-Alcohols on the properties of asphaltenes at Toluene/Air and Toluene/Water interfaces. *Colloids Interfaces* **2**(2), 3 (2018).

[35] Ahmadi, B., Molaei, A. H., Sahraei, E. & Mohammadi, A. H. Evaluation of competitive and synergistic effects of potential determining ions on interfacial tension reduction and wettability alteration in carbonate oil reservoirs. *Colloids Surf. A*. **713**, 136474 (2025).

[36] Ghorbani, M., Gandomkar, A. & Honarvar, B. Experimental investigation of asphaltene content effect on crude Oil/CO. *Minimum Miscibility Press. Period Polytech. Chem. Eng.* **64** (4), 479–490 (2020).

[37] Gandomkar, A., Torabi, F. & Nasriani, H. R. Decreasing asphaltene precipitation and deposition during immiscible gas injection via the introduction of a CO_2_-Soluble asphaltene inhibitor. *SPE Journal*. **28**(05), 1–13 (2023).

[38] Rahimi, R., Saeedi Dehaghani, A. H. & Najafi, H. A study on asphaltene adsorption onto two mineral adsorbents in the presence and absence of anionic and ionic inhibitors. *Energy Sources Part. A*. **47** (1), 2222–2238 (2020).

[39] Shadervan, A., Jafari, A., Teimouri, A., Gharibshahi, R. & Dehaghani, A. H. S. Mechanistic Understanding of asphaltene precipitation and oil recovery enhancement using SiO_2_ and CaCO_3_ nano-inhibitors. *Sci. Rep.* **14** (1), 15249 (2024). 38956269 10.1038/s41598-024-65995-1 PMC11220011

[40] Farahabadi, Z. T. & Lashkarbolooki, M. Effect of CO_2_ on the interfacial tension and swelling of crude oil during carbonated water flooding. *J. Pet. Explor. Prod. Technol.* **13**, 353–364 (2023).

[41] Hamidian, R., Lashkarbolooki, M. & Hezave, A. Z. Interfacial tension and contact angle of asphaltenic and resinous model oil in the presence of binary salts mixtures. *Sci. Rep.* **14**, 18018 (2024). 39097601 10.1038/s41598-024-68740-w PMC11297926

[42] Kashiri, R., Garapov, A. & Pourafshary, P. Effect of pH on the dominant mechanisms of oil recovery by low salinity water in fractured carbonates. *Energy Fuels*. **37**, 10951–10959 (2023).

[43] Shams, S. M., Dehghan, A. A., Kazemzadeh, Y. & Riazi, M. Experimental investigation of emulsion formation and stability: comparison of low salinity water and smart water effect. *J. Dispersion Sci. Technol.* **45**, 1646–1655 (2024).

[44] Villero-Mandon, J., Askar, N., Pourafshary, P. & Riazi, M. Importance of Fluid/Fluid interactions in enhancing oil recovery by optimizing Low-Salinity waterflooding in sandstones. *Energies* **17**, 1073 (2024).

[45] Villero-Mandon, J., Pourafshary, P. & Riazi, M. Oil/Brine screening for improved Fluid/Fluid interactions. *Colloids Interfaces*. **8** (2), 23 (2024).

[46] Zivar, D., Ishanov, A. & Pourafshary, P. Insights into wettability alteration during low-salinity water flooding by capacitance-resistance model. *Pet. Res.* **7**, 500–510 (2022).

[47] Seidy-Esfahlan, M., Khodapanah, E. & Tabatabaei-Nezhad, S. A. Production improvement mechanisms in combined low salinity water flooding and preformed particle gel treatment. *Results Eng.* **22**, 102126 (2024).

[48] Zapata, Y. et al. Well-based monitoring of CO_2_ geological sequestration operations in saline aquifers: critical insights into key questions. *Carbon Capture Sci. Technol.* **5**, 100079 (2022).

[49] Suramairy, R. et al. Impact of reservoir organic acid and Brine salinity on CO_2_-rock interfacial tension and wettability in carbonate rocks: insights for geological CO_2_ storage. *Results Eng.* **27**, 105983 (2025).

[50] Iranfar, S., Sadeghpour, F., Khaksar-Manshad, A., Naderi, M. & Shakiba, M. An Eigenvalue-Driven framework for the ranking and selection of optimal geological CO_2_ storage sites. *Results Eng.* **27**, 106770 (2025).

[51] Tavakkoli, M. et al. Effect of emulsified water on asphaltene instability in crude oils. *Energy Fuels*. **30**, 3676–3686 (2016).

[52] Lu, R., Lai, L. & Zhang, H. Stabilization mechanism of emulsion gels of crude oil with low asphaltene, resin, and wax contents. *J. Mol. Liq*. **417**, 126496 (2025).

[53] Peng, Y. et al. Effect of asphaltenes on the stability of water in crude oil emulsions. *Materials* **18**, 630 (2025). 39942296 10.3390/ma18030630 PMC11818801

[54] Yaseen, S. & Mansoori, G. A. Asphaltene aggregation onset during high-salinity waterflooding of reservoirs (a molecular dynamic study). *Pet. Sci. Technol.* **36**, 1725–1732 (2018).

[55] Mokhtari, R., Ayatollahi, S. & Fatemi, M. Experimental investigation of the influence of fluid-fluid interactions on oil recovery during low salinity water flooding. *J. Pet. Sci. Eng.* **182**, 106194 (2019).

[56] Shahsavani, B., Riazi, M. & Malayer, M. R. Asphaltene instability in the presence of emulsified aqueous phase. *Fuel* **305**, 121528 (2021).

[57] Tajikmansori, A., Saeedi-Dehaghani, A. H., Sadeghnejad, S. & Haghighi, M. New insights into effect of the electrostatic properties on the interfacial behavior of asphaltene and resin: an experimental study of molecular structure. *J. Mol. Liq*. **377**, 121526 (2023).

[58] Lake, L. W., Lotfollahi, M. & Bryant, S. L. Chapter 2-CO_2_ enhanced oil recovery experience and its messages for CO_2_ storage. in Science of Carbon Storage in Deep Saline Formations (eds Newell, P. & Ilgen, A.) G.) 15–31 (2019).

[59] Soleymanzadeh, A., Yousefi, M., Kord, S. & Mohammadzadeh, O. A review on methods of determining onset of asphaltene precipitation. *Pet. Explor. Prod. Technol.* **9**, 1375–1396 (2019).

[60] Peysson, Y., Andre, L. & Azaroual, M. Well injectivity during CO_2_ storage operations in deep saline aquifers–Part 1: experimental investigation of drying effects, salt precipitation and capillary forces. *Int. J. Greenh. Gas Control*. **22**, 291–300 (2014).

[61] Wang, P. et al. Comparative analysis of CO_2_, N_2_, and gas mixture injection on asphaltene deposition pressure in reservoir conditions. *Energies* **11**, 2483 (2018).

[62] Cho, J., Kim, T. H., Chang, N. & Lee, K. S. Effects of asphaltene deposition-derived formation damage on three-phase hysteretic models for prediction of coupled CO_2_ enhanced oil recovery and storage performance. *J. Pet. Sci. Eng.* **172**, 988–997 (2019).

[63] Hajiabadi, S. H., Bedrikovetsky, P., Borazjani, S. & Mahani, H. Well injectivity during CO_2_ geosequestration: A review of Hydro-Physical, Chemical, and Geomechanical effects. *Energy Fuels*. **35**, 9240–9267 (2021).

[64] Yusof, M. A. M. et al. Experimental study of CO_2_ injectivity impairment in sandstone due to salt precipitation and fines migration. *Pet. Explor. Prod. Technol.* **12**, 2191–2202 (2022).

[65] Razghandi, M., Madani, S. A., Ghotbi, C., Ayatollahi, S. & Dabir, B. A. bdolhossein Hemmati-Sarapardeh. *Geoenergy Sci. Eng.* **237**, 212819 (2024).

[66] Sokola, P. et al. Quantitative assessment of ceramic suspension stability using a lumisizer analytical centrifuge. *Ceramics* **8** (3), 115 (2025).

[67] Santos, D. et al. Study of asphaltene precipitation in crude oils at desalter conditions by Near-Infrared spectroscopy. *Energy Fuels*. **31** (5), 5031–5036 (2017).

[68] MacMillan, D., Tackett-Jr, J. E., Jessee, M. A. & Monger-McClure T.G. A unified approach to asphaltene precipitation: laboratory measurement and modeling. *J. Petrol. Technol.* **47**, 788–793 (1995).

[69] Ratnakar, R., Mantilla, C. & Dindoruk, B. Experimental investigation of the effects of asphaltene stability on interfacial behavior of live-reservoir-fluid systems. *SPE J.* **24**, 21–31 (2019).

[70] Yonebayashi, H. et al. Determination of asphaltene-onset pressure using multiple techniques in parallel. *SPE Prod. Oper.* **33**, 486–497 (2018).

[71] Burke, N., Hobbs, R. E. & Kashou, S. F. Measurement and modeling of asphaltene precipitation (in-cludes associated paper 23831). *J. Petrol. Technol.* **42**, 1440e1446 (1990).

[72] Jamaluddin, A. et al. Laboratory techniques to measure thermodynamic asphaltene instability. *J Can. Pet. Technol*. **41**(07). (2002).

[73] Gandomkar, A. & Nasriani., H. R. The role of direct asphaltene inhibitors on asphaltene stabilization during gas injection. *Fuel* **282**, 118827 (2020).

[74] Gandomkar, A., Nasriani, H. R., Enick, R. M. & Torabi, F. The effect of CO_2_-philic thickeners on gravity drainage mechanism in gas invaded zone. *Fuel* **331**, 125760 (2023).

[75] Cao, M., Han, C., Wang, S. & Chen, Y. Investigation of density, viscosity and derived thermodynamic properties of CO_2_-free and CO_2_-loaded poly(ethylene imine) aqueous systems at different temperatures and 0.1 MPa. *J. Mol. Liq*. **377**, 121523 (2023).

[76] Badarlis, A., Pfau, A. & Kalfas, A. Measurement and evaluation of the gas density and viscosity of pure gases and mixtures using a micro-cantilever beam. *Sensors* **15**, 24318–24342 (2015). 26402682 10.3390/s150924318 PMC4610587

[77] Gandomkar, A., Torabi, F. & Enick, R. M. Enhanced oil recovery via dissolution of low molecular weight PDMS in CO_2_ during immiscible gas injection in matrix-fracture system. *Chem. Eng. Res. Des.* **203**, 18–28 (2024).

[78] Rostami, P., Mehraban, M. F., Sharifi, M., Dejam, M. & Ayatollahi, S. Effect of water salinity on oil/brine interfacial behaviour during low salinity waterflooding: A mechanistic study. *Petroleum* **5**, 367–374 (2019).

